# Carbon Nanotube Elastic Fabric Motion Tape Sensors for Low Back Movement Characterization

**DOI:** 10.3390/s25123768

**Published:** 2025-06-17

**Authors:** Elijah Wyckoff, Sara P. Gombatto, Yasmin Velazquez, Job Godino, Kevin Patrick, Emilia Farcas, Kenneth J. Loh

**Affiliations:** 1 Active, Responsive, Multifunctional, and Ordered-materials Research (ARMOR) Laboratory, Department of Structural Engineering, University of California San Diego, La Jolla, CA 92093, USA; ewyckoff@ucsd.edu; 2 Doctor of Physical Therapy Program, San Diego State University, San Diego, CA 92182, USA; sgombatto@sdsu.edu; 3 School of Exercise and Nutritional Sciences, San Diego State University, San Diego, CA 92182, USA; yvelazquez3@sdsu.edu; 4 Qualcomm Institute, University of California San Diego, La Jolla, CA 92093, USA; jobg@fhcsd.org (J.G.); kpatrick@ucsd.edu (K.P.); 5 Laura Rodriguez Research Institute, Family Health Centers of San Diego, San Diego, CA 92105, USA; 6 School of Public Health, University of California San Diego, La Jolla, CA 92093, USA

**Keywords:** graphene, human performance, movement, nanocomposite, physical therapy, posture, skin, strain, textile, training, wearable sensor

## Abstract

Monitoring posture and movement accurately and efficiently is essential for both physical therapy and athletic training evaluation and interventions. Motion Tape (MT), a self-adhesive wearable skin-strain sensor made of piezoresistive graphene nanosheets (GNS), has demonstrated promise in capturing low back posture and movements. However, to address some of its limitations, this work explores alternative materials by replacing GNS with multi-walled carbon nanotubes (MWCNT). This study aimed to characterize the electromechanical properties of MWCNT-based MT. Cyclic load tests for different peak tensile strains ranging from 1% to 10% were performed on MWCNT-MT made with an aqueous ink of 2% MWCNT. Additional tests to examine load rate sensitivity and fatigue were also conducted. After characterizing the properties of MWCNT-MT, a human subject study with 10 participants was designed to test its ability to capture different postures and movements. Sets of six sensors were made from each material (GNS and MWCNT) and applied in pairs at three levels along each side of the lumbar spine. To record movement of the lower back, all participants performed forward flexion, left and right bending, and left and right rotation movements. The results showed that MWCNT-MT exceeded GNS-MT with respect to consistency of signal stability even when strain limits were surpassed. In addition, both types of MT could assess lower back movements.

## 1. Introduction

Piezoresistive materials exhibit a change in electrical resistance when subjected to mechanical deformation, making them viable for strain sensing applications. This property arises from alterations in their internal electronic structure, percolation pathways, and interparticle tunneling effects [1,2,3,4]. This change in resistance directly correlates to applied mechanical strain. Because of this property, conductive nanomaterials such as graphene nanosheets (GNS), single-walled carbon nanotubes (SWCNT), and multi-walled carbon nanotubes (MWCNT) are often employed for nanocomposite strain sensors [5,6,7]. The performance of piezoresistive materials is governed by parameters such as gage factor (GF), conductivity, mechanical flexibility, and strain range. GF, defined as the relative change in resistance per unit strain, is a measure of the sensor’s sensitivity. Traditional metallic strain gages exhibit low GFs of below 5, whereas nanoparticle-based sensors can achieve gage factors two orders of magnitude and higher [8]. With highly elastic substrates, the GF has been shown to not only increase, but high levels of strain can be measured as well. This is evidenced by a GF of 14.9 in the elastomer polydimethylsiloxane at strains reaching over 50%, achieved with CNT as the nanomaterial [9]. GNS has been applied to flexible tape and has shown an even higher GF of over 70 under strain levels of ~10% [10].

Applying piezoresistive materials to films has demonstrated their potential for use in wearable applications due to their mechanical flexibility. Traditional wearable systems, such as surface electromyography (sEMG) and inertial measurement units (IMUs), have been shown to provide accurate insights into movement and muscle activity [11,12]. However, their high initial cost, design constraints, form factor, and restricted accessibility prevent their widespread use in free living environments. Integrating SWCNTs or MWCNTs in elastic materials (such as polymers and textiles) is an effective way to create sensors for characterizing human movements, and their direct application on skin prevents movement artifacts from affecting measurement accuracy [13,14,15]. Unlike traditional rigid sensors, the material in these flexible strain sensors conforms to the body’s natural curves without impeding motion. This is necessary for assessing the multi-segmental spine and has demonstrated success in applications such as posture and movement monitoring during functional tasks and daily living activities [16,17,18]. To ensure accurate and reliable measurements in these contexts, sensors must consistently translate mechanical deformation into measurable electrical signals. For piezoresistive wearables, the strain range over which the selected material maintains a linear, and more importantly reversible, response is critical for high-strain measurements [19]. This property is required for applications in physical therapy and athletic training, where detection of both small movements and large excursions is necessary, and signal loss due to inconsistent properties or irrecoverable resistance values may occur.

Due to the requirements for both sensitivity and range of strain measures for clinical assessments of low back movement, and the limitations of existing portable sensors, there is a need for a compact, wireless, wearable device that could accurately monitor low back posture and movement in both clinical and everyday settings. Motion Tape (MT) is a flexible fabric-based sensor that is designed to be disposable (low cost) and directly affixed onto skin [10]. The first generation of MT was fabricated with a GNS dispersion, using commercial kinesiology tape (k-tape) as a self-adhesive substrate. GNS-MTs were tested on the shoulder, ankle, calves, biceps, and lower back, among others, during physical training exercises [20,21,22]. GNS-MTs were validated for accurately measuring skin-strains during a variety of lower back movements, and the results were highly correlated with marker-based motion capture (mocap) measurements, even in high tension movements (i.e., flexion, rotation, and contralateral in lateral bending) [23]. However, unfilterable noise and excessive resistance values resulted in a 13.9% sensor failure rate during a previous clinical study. In forward flexion, which was the movement that produced the highest tensile skin-strains, 41.7% of GNS-MT sensors failed during a particular study [23].

Given the limitations observed with GNS-MT, an alternative piezoresistive material, MWCNT, was investigated for fabricating Motion Tapes that were less susceptible to failure resulting from high levels of applied tensile strains. This study aimed to characterize the electromechanical properties of MWCNT-MT by assessing its strain response, consistency, durability, and suitability for physical therapy applications.

## 2. Methods

Controlled mechanical loading tests and human subject measures were used to determine MWCNT-MTs’ potential for reliable, long-term use in lower back movement monitoring applications. The first set of experiments was designed to characterize the strain response of MWCNT-MTs under controlled mechanical loading conditions. Specifically, the objective was to investigate: peak strain values; the consistency of peak strain values across multiple cycles; the linearity of sensor responses to applied strain; and the effects of loading rate and fatigue. This was accomplished using a load frame that applied user-defined tensile cyclic strain patterns.

To augment the results collected from the load frame experiments, practical application of MWCNT-MT was conducted and compared to a previous GNS-MT human subject study. The study measured specific movement patterns from six MTs attached to the lower back [23]. Mocap data was recorded from retroreflective markers used to create a previously validated multi-segmental spine model for lumbar spine posture and movement [24]. Resistance data captured from GNS-MT were highly correlated with mocap results, with correlation coefficient (*r*) values ranging from 0.62 to 0.94 for forward flexion, lateral bends, and rotations [23]. Because the aforementioned study by Lee et al. [23] used GNS-MT on 10 participants, the same protocol was repeated with another set of 10 participants but with MWCNT-MT for comparison. A summary of the experimental design is included in Table 1.

### 2.1. Motion Tape Fabrication

**Figure 1:**
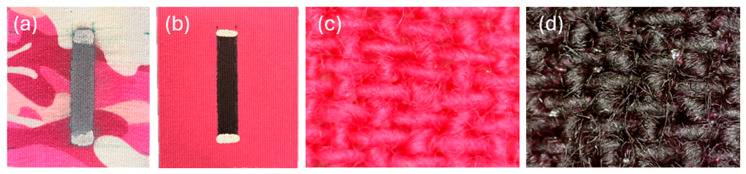
Comparison of MT made with (**a**) GNS ink and (**b**) MWCNT ink. Microscope images of the (**c**) bare k-tape and (**d**) MWCNT film deposited onto k-tape (i.e., MWCNT-MT).

GNS-MT and MWCNT-MT were fabricated following the method described by Lin et al. [10]. For each case, the sensing elements were designed by masking specific regions of the k-tape (Rock Tape, Durham, NC, USA) and adding conductive electrodes for electrical resistance measurements. For GNS-MT, the sensing element was fabricated by spray-coating a GNS-based ink onto the k-tape substrate. The ink was prepared by dispersing graphene in a solution of ethyl cellulose and ethanol. The k-tape was masked to define a 7.5 × 40 mm^2^ sensing area, and the solution was spray-coated three consecutive times to ensure uniform coating and the formation of a fiber-integrated nanocomposite. The resulting fiber-integrated nanocomposite had an initial unstrained resistance ($R_{0}$) of approximately 10 kΩ. After drying, conductive silver paste was applied at both ends of the coated area, and multistrand wires were soldered to both electrodes. The final appearance of GNS-MT is shown in Figure 1a.

For MWCNT-MT, a 2% MWCNT aqueous dispersion was used to create the sensing element. In general, a single-walled carbon nanotube physically represents a cylinder of a rolled graphene sheet. On the other hand, MWCNTs physically represent multiple, concentric SWCNTs of different diameters nested concentrically within one another. The k-tape substrate was first treated with ethanol to ensure adhesion of the first layer of the MWCNT film. The MWCNT solution was drop-cast and brushed onto the masked sensing area in four successive layers. Each layer consisted of 0.25 mL for a total of 1 mL of the MWCNT dispersion. The MWCNTs formed a tangled and percolated network of electrically conductive pathways in the form of a thin coating, adhering to the textile fibers. The MWCNT network stabilized as the sensing element fully dried over ~12 h, after which conductive silver paste (Conductor 3 Silver Ink from Voltera, Kitchener, ON, CA) was applied to both ends of the nanocomposite. Multi-strand wires (from Digi-Key, Thief River Falls, MN, USA) were soldered using low-temperature solder wire (Sn42 Bi57 Ag1 from Voltera, Kitchener, ON, Canada). The final MWCNT film had an *R*_0_ of approximately 1 kΩ. A picture of the MWCNT-MT sensor is shown in Figure 1b. In addition, a high-resolution digital microscope image of the bare k-tape is shown in Figure 1c. It can be seen from Figure 1d that the MWCNT solution is well integrated with the textile substrate. Instead of forming a continuous film across the substrate surface, the MWCNT solution uniformly coats all the individual textile fibers.

### 2.2. Tensile Loading Experimental Design

**Figure 2:**
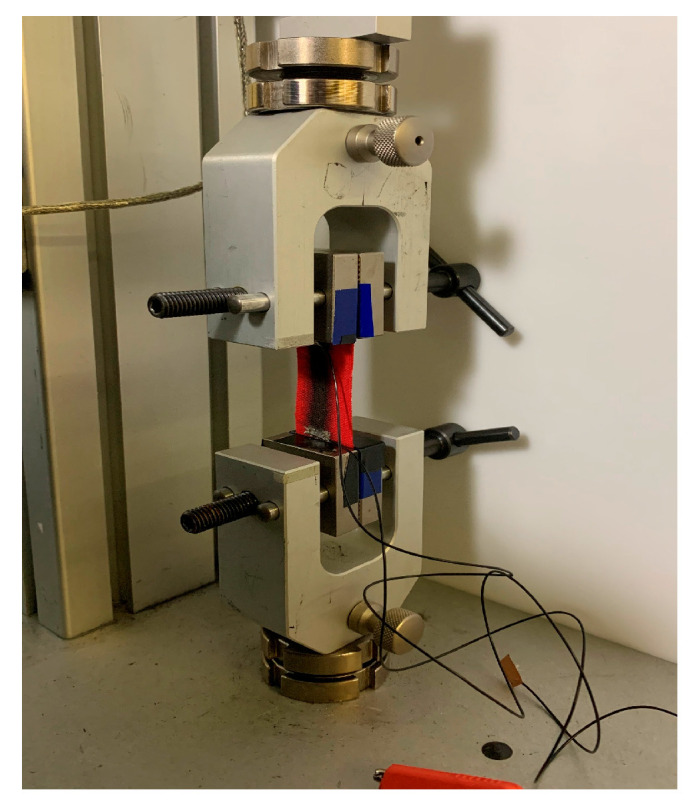
The tensile loading experiment for sensor characterization included applying strain to MT in the longitudinal direction in a Test Resources load frame.

The electromechanical properties of the MWCNT-MT were validated using a load frame shown in Figure 2 (100R from Test Resources, Shakopee, MN, USA) to apply cyclic uniaxial tensile strains. Resistance was recorded using a digital multimeter (34401A from Keysight, Santa Rosa, CA, USA). Displacement-controlled, uniaxial tensile tests were performed on bare rectangular (40 × 60 mm^2^) k-tape with sensing regions (7.5 × 40 mm^2^) specimens using the load frame. Two-cycle tests were performed to peak strains of 1% to 10% at a constant loading–unloading rate of $\frac{\varepsilon_{max}}{4}$ mm s^−1^, where ε represents strain, and $\varepsilon_{max}$ is the peak strain value for each test. Thus, all tests had the same duration. The maximum value of 10% was chosen based on the strain range of the previous version of GNS-MT. The electrical resistance of each specimen was recorded using the multimeter sampling at ~1.5 Hz. Specimen dimensions were measured using a digital caliper (NEIKO, Corona, CA, USA).

### 2.3. Human Movement Pattern Experimental Design

**Figure 3:**
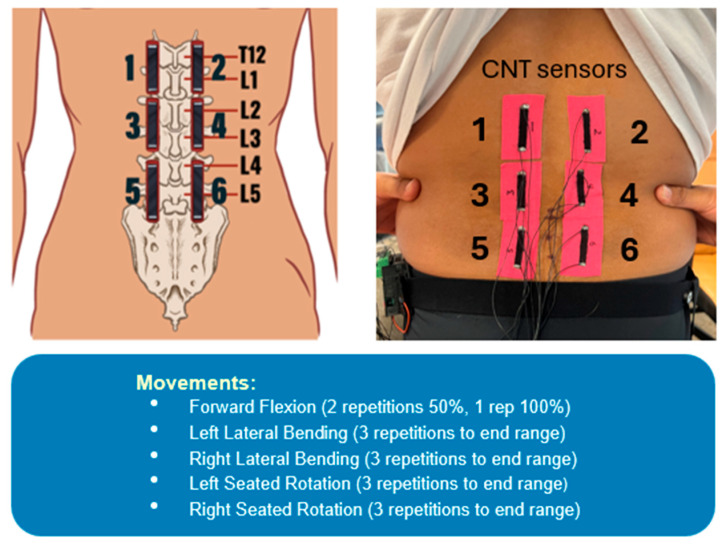
The schematic (**left**) and picture (**right**) show MT sensor numbers and their corresponding locations on the lower back. A list of lower back movements conducted during the human subject study is also provided.

To evaluate MT performance in human subjects in a controlled laboratory setting, an experiment was designed to measure strain response during specific low-back movements and to compare signal quality of MWCNT-MT with that from GNS-MT from the prior study. For testing with each material, six MT sensors were created and placed at three levels on each side of the lower back (Figure 3). Placement started with the middle sensors (Sensors 3 and 4), with the bottom edges aligned just above the L4 spinous process. This was intended to cover the L2–L3 and L3–L4 junctions in most cases. The upper Sensors 1 and 2 and lower Sensors 5 and 6 were placed directly above and below the middle sensors, with minimal separation between the top and bottom of the k-tape. This can also be seen in Figure 3, where corresponding lumbar spine locations are provided alongside sensor positions. For testing with each material, 10 participants performed a series of controlled lower back movements, including lateral bending to each side, rotation to each side, and forward flexion, with each movement repeated three times. All movements were performed to maximum range of motion, except forward flexion. Forward flexion was limited to 50% of end range for two repetitions, then full range of motion for the final repetition. A summary of the experiment is provided in Figure 3. Although the effect of humidity on MWCNTs is well-documented [25,26], this study was conducted indoors under controlled environmental conditions. Thus, the effects of ambient temperature and moisture were not factored into the analysis. It should be mentioned that current work is also in progress to investigate the effects of temperature on sensor performance and how ambient effects could be automatically compensated [27].

The data acquisition (DAQ) system used in these experiments transmitted resistance measurements wirelessly using Bluetooth Low Energy (BLE). The DAQ was composed of an ESP32 microcontroller and an analog-to-digital converter (ADC) from Analog Devices, Wilmington, MA, USA. Resistance data were saved to a spreadsheet and then processed by a custom MATLAB R2023a program.

### 2.4. Participants Recruitment

Data were first collected with GNS-MT [23] on 10 participants (5 males, 5 females; ages: 22.4 ± 2.1 years), then repeated with an additional 10 participants using MWCNT-MT (5 males, 5 females; ages: 39.6 ± 17 years). A total of 20 participants without low-back pain were tested to ensure full ranges of lower back movement could be assessed. This sample size was deemed sufficient for preliminary clinical assessments of MT for measuring lower back strain. It provides enough variability in quantitative measures to inform device refinement and future studies with larger populations.

Recruitment efforts included posting flyers throughout the San Diego area and on social media. Eligibility screening was conducted through an online questionnaire. Participants met the inclusion criteria if they: (1) were between 18 and 65 years old, (2) had no history of low-back pain in the past year (i.e., no chronic low-back pain or CLBP), and (3) could follow basic movement instructions in English or Spanish. Exclusion criteria included the inability to perform basic trunk movements (e.g., bending forward or sideways) and functional activities (e.g., walking and stair climbing). The studies using different MTs (GNS and MWCNT) were approved by the Institutional Review Board at San Diego State University (HS-2022-0269, HS-2024-0217), and all participants provided informed consent before participation.

## 3. Results

### 3.1. Strain Response Characterization

Four MWCNT-MT samples were prepared, and each sample was first subjected to two-cycle tensile loading to evaluate their electromechanical response across the strain range of 1% to 10%.

**Figure 4:**
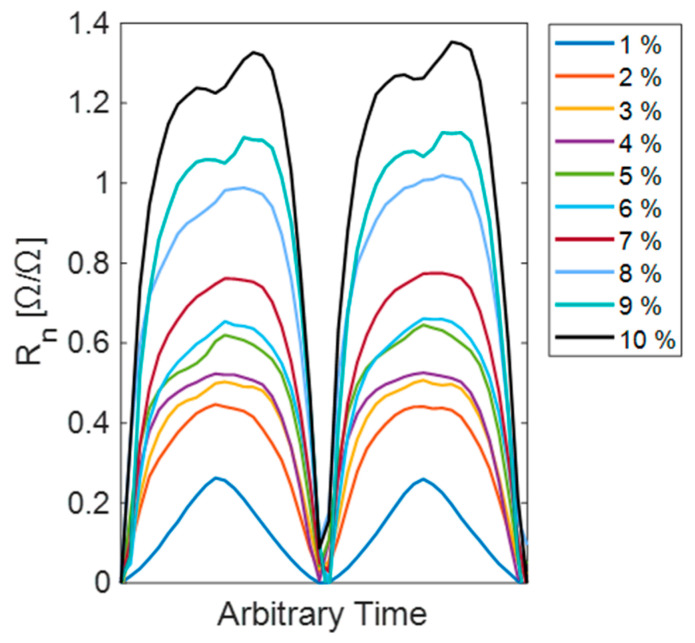
Two-cycle results of resistance response up to a maximum strain of 10% for MWCNT.

**Resistance response to strain**. Figure 4 presents two-cycle results for one specimen. First, the normalized resistance values ($R_{n}$) are calculated from Equation (1). $R_{0}$ corresponds to the baseline resistance of MT in an unstretched position, while *R* is the electrical resistance of Motion Tape measured at any instance in time.

(1) $$R_{n}=(R{-R}_{0})/R_{0}$$

Peak strain levels are also labeled for each line plot. According to Equation (1), the magnitude of $R_{n}$ is influenced by the baseline resistance. This can vary slightly between samples due to fabrication differences. For the results presented in Figure 4, $R_{0}$ was around 3.5 kΩ and the magnitude of $R_{n}$ was lower than in subsequent experiments with MWCNT-MT, which have a lower $R_{0}$. This figure serves as an initial characterization of sensor behavior and helped to inform selection criteria for ideal baseline resistance in later testing. An $R_{0}$ range of 0.6–1 kΩ was then chosen as the target to increase $R_{n}$ (and thus sensitivity) while maintaining robustness to strain.

The results from Figure 4 imply that the nonlinear response of the MWCNT-MT sample begins around 2% strain, with the characteristic double-peaking behavior appearing around 9% strain. The nonlinear response of CNT-based strain sensors at higher applied strains have been observed experimentally and numerically [28,29,30] and may be attributed to network rearrangement or release of strain energy from the breaking of CNT junctions [9]. This behavior necessitates the modeling of the sensor’s nonlinear response to accurately interpret strain from resistance changes, particularly above 1% strain where simple linear approximations are no longer valid. These results are reported later in Section 3.2.

**Sensor consistency across samples.** To compute the consistency in sample preparation, standard deviation in the peaks of the normalized resistance at each strain level was calculated. This is intended to quantify the consistency of the signal at each peak strain. Standard deviation is calculated for peak values of $R_{n}$ across samples at the same peak strain, as a consistent sensor response should yield similar normalized resistance changes for the same applied strain. These results are provided in Table 2, where 8% strain is selected as the maximum based on the responses observed in Figure 4.

**Figure 5:**
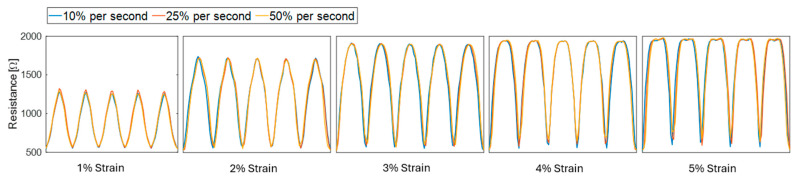
Response of MWCNT-MT to three different loading rates at 1–5% strain.

**Strain rate sensitivity and signal stability under repeated loading.** To assess the impact of strain rate and repeated loading on sensor consistency, additional cyclic electromechanical tests were performed at three different loading rates (10%, 25%, and 50%) of maximum strain per second on a new sample with an $R_{0}$ of about 550 Ω. Each test was repeated for five cycles within the 1–5% strain range, to evaluate how loading rate influences signal response. A representation of these results is provided in Figure 5, where each dataset is interpolated to normalize the time series for comparison of the responses. $R_{0}$ was subtracted from the dataset to align the plots from zero.

Figure 5 depicts that strain rate had a minimal effect on the resistance response of the sensors. This is evidenced by standard deviations of 3.18 Ω to 24.26 Ω (0.16% to 1.90%) in each cycle’s peak for the varied load rates across all strains, where the highest standard deviation was for 1% strain. The introduction of nonlinearity is also observable across increasing strain magnitudes.

**Figure 6:**
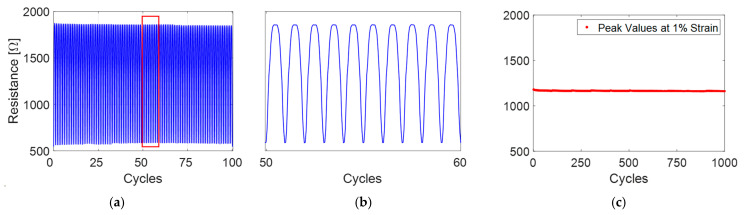
The resistance time history response for MWCNT-MT under (**a**) 100 cycles of loading at 4% peak strain is plotted, and a red rectangle section of the time history was randomly selected to show (**b**) a zoomed-in view from cycles 50 to 60. (**c**) The resistance values corresponding to 1% peak strains during 1000 cycles of loading are plotted to show slight drift.

In a separate experiment, cyclic loading at a constant rate was extended to 100 cycles from 1% to 5% strain to assess signal stability over time. The number of cycles was selected as a representative benchmark to evaluate signal stability within the expected lifespan during short-term monitoring applications, as the sensors are designed to be disposable. However, a 1000 cycle test at 1% strain was also performed to characterize drift in the sensor response over extended use. A 100-cycle response is shown for 4% strain in Figure 6a, with a zoomed-in view given in Figure 6b. The peak values for the 1% strain 1000 cycle test are shown in Figure 6c. The same y-axis scale is used for Figure 6a–c to highlight the minimal drift in peak resistance values over 1000 cycles. This test was designed to simulate prolonged sensor use under repeated mechanical strain. Stress affects both the MWCNT conductive network and the physical deformation of the substrate, making it necessary to evaluate signal stability over extended cyclic loading.

Overall, the electromechanical response across 100 cycles for each strain rate demonstrated high signal stability, with resistance changes closely tracking the applied strain. The signal consistently returns near baseline after each cycle, indicating minimal drift and effective recovery of the MWCNT conductive network. Notably, the response was observed to stabilize to a consistent baseline after ~30 cycles, suggesting that there are initial settling effects that may be due to stress relaxation of the k-tape matrix. However, peaks remained constant after ~3 cycles. For the 100-cycle tests, standard deviations ranged from 3.10 Ω to 7.95 Ω (0.24% to 0.46%) in cyclic peaks with the highest value occurring in the 3% strain response. To quantify signal drift across cycles, linear least-squares regression was performed on the peak resistance values. The slope of the fitted linear line represents the rate of change in peak resistance over time (i.e., with respect to the number of cycles). Each response demonstrated near-zero slope, with a maximum slope of −0.028 in the 1% strain response. For the 1000-cycle test at 1% peak strain, the slope of the fitted line was even smaller at −0.007, with a maximum change in peak electrical resistance of 7.87 Ω (i.e., 0.67%). These results indicate high signal stability for MWCNT-MT.

### 3.2. Nonlinear Fit Modeling

**Figure 7:**
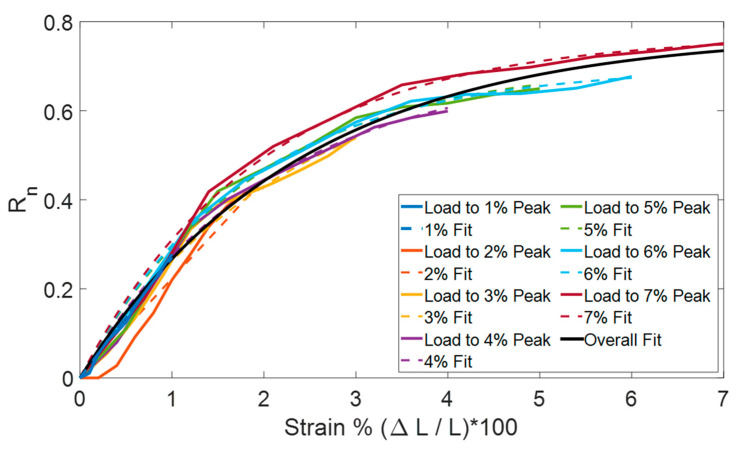
Nonlinear regression curves were fitted to the normalized change in resistance data with respect to the applied strains to model MWCNT-MT strain responses.

It can be observed in Figure 4 that the MWCNT-MT response to strain is only linear at 1% strain. Thus, MWCNT-MT resistance change was modeled using an exponential saturation function to capture its electromechanical response’s sharp initial increase and subsequent plateauing. First, the normalized resistance values ($R_{n}$) are calculated (Equation (1)). Next, the model to fit the data was calculated as

(2) $$R_{n,fit}=a*(1-e^{-b*\varepsilon }),$$

where the fitting parameter a corresponds to the maximum asymptotic response and b governs the rate at which the response reaches saturation. The applied strain is represented by ε. Applied strain is labeled as percent strain in Figure 7, where the best-fit nonlinear regression curves ($R_{n,fit}$) for the MWCNT-MT sample were overlaid on the experimental data ($R_{n}$). Each $R_{n}$ curve represents the load profile from baseline (0% strain) up to the indicated peak strain (‘Load to 1–7% Peak’). The maximum of 7% strain was selected for the overall fit because the response curves began to diverge noticeably in shape at 8% and beyond. The figure is not intended to serve as a universal calibration curve but rather to visualize the nonlinear sensor behavior at different strain amplitudes. The predictive model (‘Overall Fit’) in Figure 7 was derived from the combined dataset across the 1–7% strain range (prior to divergence) and is presented as a representative curve characterizing the nonlinear strain response of the MWCNT-MT.

This provides a functional representation of the sensor’s nonlinear behavior under applied strain. Taking the initial slope of the overall fit yielded a strain sensitivity or gage factor of 32.74 at 20% divergence from the overall fit (~1% strain), calculated by Equation (3). The initial slope (a∗b) corresponds to the derivative of the function from Equation (2) at 0% strain.

(3) GF=a∗b/ε

While Figure 7 presents individual fits for loading to different peak strains, in practical applications, strain estimation would rely on a single unified model. The predictive curve generated by combining the datasets up to 7% strain provides a global fit that can be used to invert $R_{n}$ values and estimate strain through the inverse of the fitted exponential function. However, full validation of this back-calculation approach remains a subject for future work. Future efforts would focus on a model to estimate strain in real-world applications, such as measuring low-back strain during body movements. In addition, methods such as pre-stretching protocols and alternative sensing region geometries for improving the linearity of the resistance response at higher strain levels will be explored.

### 3.3. Human Lower Back Movement Measurment Comparison of GNS-MT vs. MWCNT-MT

**Figure 8:**
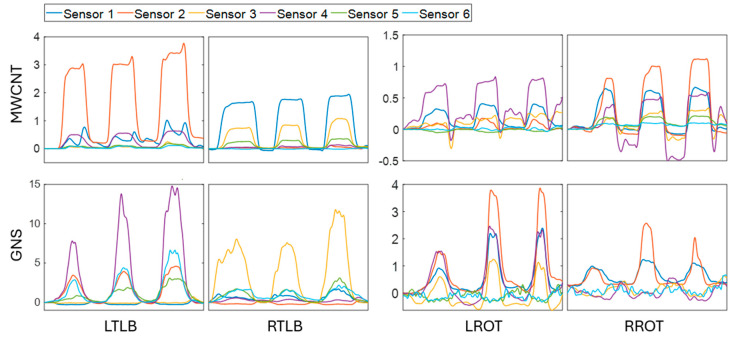
Representative normalized resistance plots for left and right trunk lateral bending (L/RTLB) and left and right rotation (L/RROT) movements by arbitrary time for MWCNT-MT (**upper**) and GNS-MT (**lower**).

To compare MWCNT-MT to GNS-MT, data were recorded and analyzed in 20 participants without low back pain, 10 for each material. Lower back strain was measured during low-back movements (i.e., lateral bends, seated rotations, and forward flexion), as outlined in Figure 3. The first set of data acquired from the previous study reflects resistance readings from MT samples prepared with GNS [23], and the second study used MWCNT-MT. Representative plots for select movements plotted as normalized resistance are provided in Figure 8. Here, $R_{n}$ was processed using a method of locally weighted scatter plot smoothing (Lowess), to reduce noise. The span for Lowess smoothing was chosen as 5%.

**Figure 9:**
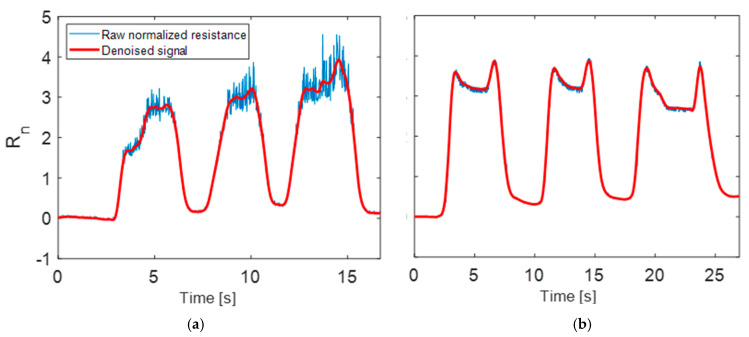
Representative plots of signal noise for Sensor 3 in forward bending for (**a**) GNS-MT and (**b**) MWCNT-MT.

Positive normalized resistance changes correspond to tensile strain in the sensing region, while small negative excursions reflect brief compression events (most notably in rotations). Despite the smoothing applied in both cases, noise can still be observed in GNS-recorded movements. Sensors 5 and 6 in rotations display noise that was not filtered out by the Lowess function. During data collection, investigators’ visual observations of the GNS-MT indicated that the sensing region was prone to crack formation under high strain and after repeated exposure to high-strain movements. As a result, significant signal noise was observed due to this disruption to the conductive pathways. The same behavior was not observed in MWCNT-MT, which appears to maintain more stable conduction pathways under loading. Noise levels were so extreme in some of the GNS-MT measurements that the signal became irrecoverable, and these signals were removed from analysis entirely [23]. An example comparison of the original and smoothed signal is provided in Figure 9 for forward bending, which was the movement with the highest magnitude of strain. Resistance recorded by Sensor 3 (middle-left sensor) was chosen as a representative signal from one subject for both GNS-MT (Figure 9a) and MWCNT-MT (Figure 9b).

Analysis was performed to calculate the signal-to-noise ratio (SNR) in each sensor for each movement to assess the signal quality of MWCNT-MT in comparison to GNS-MT. SNR was determined by first smoothing the resistance signal, then computing the noise as the difference between the raw, unfiltered data and the smoothed signal. The ratio of the power of the smoothed signal to the power of the noise component provides a direct measure of signal quality, calculated by

(4) $$SNR=10*\log_{10}\frac{\sum_{n=1}^{N}{R_{smooth}}^{2}}{\sum_{n=1}^{N}{\left(R_{raw}-R_{smooth}\right)}^{2}}$$

These values are calculated for each of the six sensors over all five movement patterns. Table 3 reports SNR values for both MWCNT-MT and GNS-MT across multiple movement sets.

The results reported in Table 3 indicate that the MWCNT-MT sensors exhibited higher signal-to-noise values across all five movements. In contrast, GNS-MT sensors displayed substantially lower values, especially for the movement with the highest strain, which was forward bending. The GNS-MT sensors were shown to display high sensitivity [21], but this comes at the cost of low SNR.

The SNR results are indicative of signal quality and each sensor’s robustness to high strains. The lowest SNRs are associated with irrecoverable noise in the GNS-MT sensors, which were identified using a threshold criterion of resistance >10 standard deviations from the mean resistance across each movement. Datasets that included irrecoverable noise were deemed unusable and considered a sensor failure, and these data were removed from the original study. 

**Figure 10:**
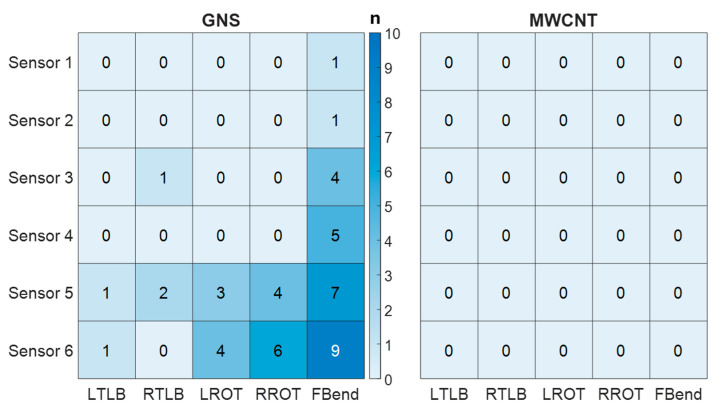
Comparison of number of subjects with sensor failures using GNS-MT and MWCNT-MT across different movement types.

To further compare the performance of GNS-MT and MWCNT-MT for measuring human movement, Figure 10 was generated as a heatmap to visualize the distribution of sensor failures across movements for both GNS-MT and MWCNT-MT. This heatmap demonstrates a significant disparity in sensor failure rates between GNS-MT and MWCNT-MT. GNS-MT sensors exhibited numerous failures, particularly in Sensors 5 and 6 during forward bending, which imparts the greatest strain on the sensors. These findings directly align with the SNR results in Table 3; Sensors 5 and 6 displayed the smallest SNRs for GNS-MT during forward bending. MWCNT-MT sensors showed zero failures across all sensor positions and movement types, suggesting that MWCNT-MT offers tremendous reliability for wearable strain sensing applications that include high-strain movements.

## 4. Discussion

### 4.1. MWCNT-MT Sensor Characterization

The plots in Figure 4 display the strain responses exhibited by MWCNT-MT sensors. The sensors demonstrated a nonlinear response beyond 2% strain, necessitating the development of an exponential saturation function to model its behavior. Despite this nonlinearity, MWCNT-MT displayed a consistent response across all strain levels. This was evidenced by an average normalized standard deviation of 0.074 (Ω/Ω) (range: 0.04–0.09 Ω/Ω) for two-cycle tests across strain levels from 1% to 8% (Table 2). Importantly, standard deviation did not increase with strain level. To capture this behavior, an exponential saturation function was developed and applied as a nonlinear fit model. This model effectively represents the observed resistance changes up to 7% strain, as shown in Figure 7.

Rate sensitivity analysis conducted at loading rates of 10%, 25%, and 50% of max strain/second (Figure 5) showed minimal influence on peak resistance. Standard deviations across cycles were between 3.90 Ω to 23.78 Ω, with the largest variability at 1% strain. The primary difference was observed in the sensor’s ability to fully return to baseline at the unstretched position following each cycle, but this may be attributed to the sampling rate of the multimeter software as the sensors consistently returned to the original baseline by the end of the full set of cycles. Another possible explanation for the incomplete return to baseline after each cycle is minor viscoelastic relaxation or mechanical settling of the k-tape substrate, which could cause a slight lag in recovery even after the external load is removed. This does not necessarily reflect failure of the sensing element itself. Signal stability under extended loading was validated through 100-cycle tests at 1–5% strain. Figure 6 reports the 4% strain response, which showed consistent return to baseline and minimal signal drift. The resistance signal demonstrated peak variability between 3.10 Ω to 7.95 Ω, and peak slope values from linear regression near zero (−0.028 to −0.002). Collectively, the characterization of MWCNT-MT suggests that the material may be suitable for human movement applications, especially with high-strain measurements.

### 4.2. Human Movement Analysis

To evaluate real-world applicability, GNS-MT and MWCNT-MT were deployed across six lumbar spine locations during five controlled lower back movements: left and right lateral bends, left and right seated rotations, and forward flexion. Each movement was tested in 10 participants for each sensor material. One issue observed in the GNS-MT from the initial study was signal quality, especially for high strains. Quantitative signal quality was assessed and compared using signal-to-noise ratios calculated from smoothed resistance signals. MWCNT-MT consistently outperformed the GNS-MT in all movement types, with more than double average SNR of 38.54 across all sensors for all movements compared to 17.62. The highest SNR values of 45.21 (LTLB) and 45.10 (RROT) were observed in Sensor 6 for MWCNT-MT. Sensors 5 and 6 were the worst performing sensors in GNS-MT and showed negative SNR in forward bending (−6.15 and −6.32, respectively).

This indication of critical signal degradation led to the development of a heatmap to visualize sensor failures for both materials, defined as irrecoverable resistance signals. The heatmap in Figure 10 revealed zero failures across all MWCNT-MT sensor positions and movement types, as compared to numerous failures in GNS-MT sensors. Most failures (27 out of 60 datasets) occurred during forward bending, which imposed the highest strains. GNS-MT Sensors 5 and 6 recorded the highest failure rates, which also correlated with their lowest SNR values. Compared to previous studies on human motion sensors, this approach recorded localized spinal strain patterns and distinct lower back movements by tracking the skin deformation itself. Previously, the high strains associated with this approach caused irrecoverable signals, but the high SNRs exhibited in MWCNT-MT demonstrate their potential for application in various dynamic movements.

## 5. Conclusions

The objective of this study was to characterize the performance of MWCNT-MT wearable sensors for assessing strain response, consistency, and durability in the context of physical therapy applications. MWCNT-MT was compared to GNS-MT to evaluate how well they characterize low-back movements. The test results showed that MWCNT-MT offers stable electromechanical behavior suitable for human movement monitoring and analysis. Controlled electromechanical cyclic loading tests demonstrated that MWCNT-MT exhibited low standard deviations across different peak strain levels, indicating consistency in signal response. Human movement analysis further revealed that MWCNT-MT sensors maintained great signal stability during all movements. The findings suggest that MWCNT-MTs could reliably capture posture and movement data even at high strains, although its electromechanical response may be nonlinear. Overall, MWCNT-MT is suitable for use in physical therapy, athletic training, and human movement monitoring applications. Its robustness to high strain states and consistent signal response provides a valuable tool for assessing lower back movements. Future work will investigate the use of MWCNT-MT in clinical applications, particularly for identifying potential issues related to lower back pain in clinical settings.

## Figures and Tables

**Table 1:** Summary of the included experimental data.

Experiment Type	Material	Objective Outcome	Reference
Load Frame Experiment	MWCNT	Sensor consistency, strain limit, and linearity analysis	Current study (new)
Human Subject Study 1 [N = 10]	GNS	Resistance during lower back movements, mocap correlation	Previous study [23]
Human Subject Study 2 [N = 10]	MWCNT	Resistance during lower back movements	Current study (new)

**Table 2:** Peak standard deviation in $R_{n}$ for MWCNT-MT.

Strain	1%	2%	3%	4%	5%	6%	7%	8%	Mean
** Peak SD (Ω/Ω ** **)**	0.042	0.074	0.090	0.074	0.084	0.094	0.045	0.090	0.074

Notably, the standard deviation remained below 0.1 and did not correlate to the level of strain. The consistency suggests that MWCNT may provide a reliable and predictable response for strain sensing applications where signal stability is critical.

**Table 3:** Sensor signal quality when subjected to low back movement patterns.

Sensor Number	MWCNT					GNS				
	Signal-to-Noise Ratio					Signal-to-Noise Ratio				
	LTLB	RTLB	LROT	RROT	FBEND	LTLB	RTLB	LROT	RROT	FBEND
MT1	36.43	37.34	36.67	36.89	32.31	35.19	25.12	27.68	33.20	16.11
MT2	42.38	36.13	39.13	37.29	31.82	27.81	34.93	27.08	29.28	14.53
MT3	30.91	37.03	38.22	39.60	29.60	31.34	17.37	19.65	24.66	4.608
MT4	41.39	35.31	40.15	41.61	31.34	16.59	29.38	22.36	18.42	1.581
MT5	43.28	44.69	42.38	43.81	35.48	17.27	11.38	4.815	4.928	−6.146
MT6	45.21	44.96	43.79	45.10	35.87	14.54	23.99	5.833	1.469	−6.321
Mean	39.93	39.24	40.06	40.72	32.74	23.79	23.70	17.90	18.66	4.062

## Data Availability

The data presented in this study are available upon request from the corresponding author. The data are not publicly available, owing to ethical concerns.
